# Distinct transcriptional programs stratify ovarian cancer cell lines into the five major histological subtypes

**DOI:** 10.1186/s13073-021-00952-5

**Published:** 2021-09-01

**Authors:** Bethany M. Barnes, Louisa Nelson, Anthony Tighe, George J. Burghel, I-Hsuan Lin, Sudha Desai, Joanne C. McGrail, Robert D. Morgan, Stephen S. Taylor

**Affiliations:** 1 Division of Cancer Sciences, Faculty of Biology, Medicine and Health, University of Manchester, Manchester Cancer Research Centre, Oglesby Cancer Research Building, 555 Wilmslow Road, Manchester, M20 4GJ UK; 2 Manchester Centre for Genomic Medicine, St Mary’s Hospital, Manchester University NHS Foundation Trust, Oxford Road, Manchester, M13 9WL UK; 3 Bioinformatics Core Facility, Faculty of Biology, Medicine and Health, University of Manchester, Michael Smith Building, Dover Street, Manchester, M13 9PT UK; 4 Department of Histopathology, The Christie NHS Foundation Trust, Wilmslow Rd, Manchester, M20 4BX UK; 5 Department of Medical Oncology, The Christie NHS Foundation Trust, Wilmslow Rd, Manchester, M20 4BX UK

**Keywords:** Ovarian cancer, Non-negative matrix factorization, RNA sequencing, Subtype classification, Machine learning, Transcriptomics

## Abstract

**Background:**

Epithelial ovarian cancer (OC) is a heterogenous disease consisting of five major histologically distinct subtypes: high-grade serous (HGSOC), low-grade serous (LGSOC), endometrioid (ENOC), clear cell (CCOC) and mucinous (MOC). Although HGSOC is the most prevalent subtype, representing 70–80% of cases, a 2013 landmark study by Domcke et al. found that the most frequently used OC cell lines are not molecularly representative of this subtype. This raises the question, if not HGSOC, from which subtype do these cell lines derive? Indeed, non-HGSOC subtypes often respond poorly to chemotherapy; therefore, representative models are imperative for developing new targeted therapeutics.

**Methods:**

Non-negative matrix factorisation (NMF) was applied to transcriptomic data from 44 OC cell lines in the Cancer Cell Line Encyclopedia, assessing the quality of clustering into 2–10 groups. Epithelial OC subtypes were assigned to cell lines optimally clustered into five transcriptionally distinct classes, confirmed by integration with subtype-specific mutations. A transcriptional subtype classifier was then developed by trialling three machine learning algorithms using subtype-specific metagenes defined by NMF. The ability of classifiers to predict subtype was tested using RNA sequencing of a living biobank of patient-derived OC models.

**Results:**

Application of NMF optimally clustered the 44 cell lines into five transcriptionally distinct groups. Close inspection of orthogonal datasets revealed this five-cluster delineation corresponds to the five major OC subtypes. This NMF-based classification validates the Domcke et al. analysis, in identifying lines most representative of HGSOC, and additionally identifies models representing the four other subtypes. However, NMF of the cell lines into two clusters did not align with the dualistic model of OC and suggests this classification is an oversimplification. Subtype designation of patient-derived models by a random forest transcriptional classifier aligned with prior diagnosis in 76% of unambiguous cases. In cases where there was disagreement, this often indicated potential alternative diagnosis, supported by a review of histological, molecular and clinical features.

**Conclusions:**

This robust classification informs the selection of the most appropriate models for all five histotypes. Following further refinement on larger training cohorts, the transcriptional classification may represent a useful tool to support the classification of new model systems of OC subtypes.

**Supplementary Information:**

The online version contains supplementary material available at 10.1186/s13073-021-00952-5.

## Background

Ovarian cancer (OC) is the most common cause of gynaecological-related cancer death in Europe and North America [1]. Although 90% of tumours are epithelial in origin, these tumours exhibit substantial heterogeneity in terms of clinical presentation and molecular biology [2]. Subclassification is therefore essential, not only to personalise treatment, but also to provide a framework to assist scientific research [3]. While various classifications have been proposed, it is now widely accepted that epithelial OC can be subdivided into five main histological types [3–6]. The predominant subtype is high-grade serous (HGSOC), which accounts for 70–80% of cases, while rarer subtypes include low-grade serous (LGSOC [<5%]), endometrioid (ENOC [10%]), clear cell (CCOC [10%]) and mucinous (MOC [3%]) [7]. Expansion of next-generation sequencing has revealed the distinct molecular characteristics of each subtype; for example, HGSOC is characterised by near-ubiquitous *TP53* mutation, germline and/or somatic mutations in genes involved in homologous recombination (HR) repair and genome-wide copy-number variation (CNV) [8–10]. Unlike HGSOC, the other subtypes are characterised by mutations in the MAPK and PI3K/AKT pathway, and while ~60% of MOC also have *TP53* mutations [11, 12], *TP53* is altered in only around 15–20% of ENOC and CCOC [13–15] and less than 10% of LGSOC [16–18].

**Fig. 1:**
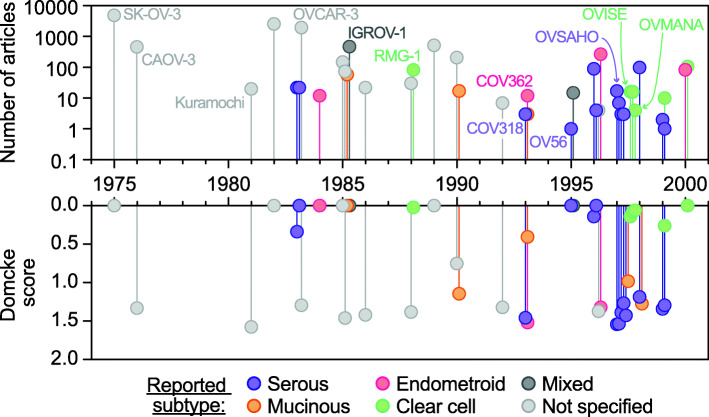
Cell line usage based on PubMed citations. Top, total number of PubMed usages of each of the 44 epithelial ovarian cancer cell lines for which RNAseq data is available within the CCLE. Bottom, HGSOC-likelihood scores as determined by Domcke et al. analysis of ovarian cancer cell lines correlated with The Cancer Genome Atlas HGSOC patient samples. Cell lines are separated along the *x*-axis based on the year of their first usage. Cell lines are coloured by the subtype of epithelial ovarian cancer reported in their primary literature source

A greater understanding of the different molecular events that underpin ovarian carcinogenesis is driving the expansion of tailored therapies [19]. Current treatment guidelines have been broadly established from studies of HGSOC [4], yet differences in chemotherapy sensitivity between subtypes highlight the need for subtype-specific research [20, 21]. Such research in turn requires appropriate model systems that robustly reflect each subtype. The Cancer Cell Line Encyclopedia (CCLE) includes over 40 OC cell lines; however, many widely used lines were established over 20 years ago, prior to the advent of current histological subclassification (Fig. 1). In addition, cell lines with a designated histotype may have genetically drifted from the original patient cells [22–24]. In a landmark study in 2013, Domcke et al. compared cell line CNV and mutation profiles with patient samples from The Cancer Genome Atlas (TCGA) to identify lines closely resembling HGSOC [9, 25]. In turn, HGSOC lines identified in this study were shown to display the profound cell division abnormalities typical of patient-derived tumour cells [26, 27]. However, the subtype of the ‘non-HGSOC’ lines was not determined by Domcke et al. Other studies have also sought to determine the subtype of OC cell lines by, for example, morphological and immunohistochemistry analysis [28, 29], and while these are largely in agreement with Domcke et al., in their identification of HGSOC cell models, non-HGSOC are often only designated as unclassified, atypical non-serous or undistinguishable between ENOC and CCOC. Thus, uncertainty remains regarding which cell lines are representative of LGSOC, ENOC, CCOC and MOC [25, 28–31]. Furthermore, while cell lines can be tractable models for research, they often underrepresent tumour heterogeneity [32]. To address this, researchers are developing living biobanks of patient samples [27, 33–36], and as the use of biobanks expands, it is important that their subtype classification can be confirmed, particularly if clinical annotation is unavailable.

To support the classification of both established cell lines and novel OC models, we aimed to develop a methodology to classify all subtypes based on molecular features. In particular, now that RNA sequencing (RNAseq) data is widely available, we sought to determine whether a transcriptional ‘fingerprint’ could distinguish subtypes in an unbiased manner. While the utility of RNAseq as a tool for developing biomarkers is in its infancy, techniques are established and are becoming more accessible and less costly. The challenge, however, is in the distilling of robust ‘fingerprints’ from these complex datasets. One approach to reduce complexity is non-negative matrix factorisation (NMF), which has been utilised to reduce the dimensionality of transcriptional profiles from thousands of genes to a subset of important metagenes, concurrently providing meaningful class discovery [37]. Here, we apply NMF to the gene expression profiles of 44 epithelial OC cell lines, recently sequenced as part of the CCLE project [38], and find that this stratifies the cell lines into five robust clusters. Subsequent cross-referencing of cell line mutational profiles against profiles from clinical cohorts confirmed that the NMF clusters represent the five main subtypes of epithelial OC. In contrast to the study by Domcke et al., this positive assignment of cell lines into subtype-specific clusters now identifies appropriate models for all five main subtypes.

Finally, we sought to translate the NMF clustering into a transcriptional classifier for novel OC models using a living biobank of patient samples. The classifier was first created by training a machine learning algorithm on the subtype metagene profiles defined by NMF of the cell lines. The potential utility of the classifier was then tested using RNAseq generated from our biobank and comparison of a predicted subtype with clinical diagnosis.

## Methods

### Literature search

A literature search was carried out to determine which of the CCLE samples were representative of the major types of epithelial OC. This eliminated three cell lines from the panel of OC cell lines: one malignant Brenner tumour and one granulosa cell tumour and an engineered cell line. The malignant Brenner tumour was removed as, although classified as an epithelial tumour type [3], it is the only cell line of this derivation and it was considered to impair consensus clustering for this reason. Usage of CCLE OC cell lines in research was determined by PubMed search using all known aliases for the cell lines. It should be noted that we only count the number of articles where the cell line is specified in the title or abstract, therefore missing literature that only specifies within the article text which cell lines were used.

### Cancer Cell Line Encyclopedia RNAseq data acquisition

Forty-four cell lines representative of the major ovarian cancer subtypes analysed by RNA sequencing as part of the CCLE were identified [38] (Additional file 1: Table S1). We obtained Raw sequence files in FASTQ format from the European Nucleotide Archive (http://www.ebi.ac.uk/ena/; accession PRJNA523380) and remapped raw sequence reads using gene annotations from Gencode v32, to enable comparison with our own RNAseq of patient-derived OCMs (see below and [27]).

### Ex vivo ovarian cancer models

Research samples were obtained with informed patient consent from the Manchester Cancer Research Centre (MCRC) Biobank. The MCRC Biobank is licensed by the Human Tissue Authority (licence number: 30004) and is ethically approved as a research tissue bank by the South Manchester Research Ethics Committee (Ref: 07/H1003/161+5). The role of the MCRC Biobank is to distribute samples and does not endorse studies performed or the interpretation of results. For more information, see https://www.mcrc.manchester.ac.uk/research/mcrc-biobank/about-the-mcrc-biobank/.

Ex vivo ovarian cancer models (OCMs) were expanded from 33 clinical specimens from 27 patients, of which 11 were published previously (Additional file 1; Table S2) [27]. Two were solid tumour specimens and 31 were isolated from ascites. Histopathological review of cases revealed 23 of 27 patients had a conclusive diagnosis consistent with WHO guidelines. Of these 23 patients, 82.6% were HGSOC (19 of 23) and 8.7% were MOC and LGSOC (each two of 23). Two patients displayed atypical morphology, one displaying moderately differentiated serous adenocarcinoma of intermediate grade and a second with possible mixed LGSOC and HGSOC features. Two further patients had a diagnosis recorded of suspicious of adenocarcinoma arising from the gynaecological tract. The average age for HGSOC was 63.9 years (standard deviation, ± 11.1), MOC 39 years (± 14) and LGSOC 48.5 years (± 7.5). The average overall survival for HGSOC was 29.8 months (± 22.9), MOC was 58.7 months (± 5.6) and LGSOC was 37.6 (± 0.6). Initially, 33 OCMs were generated; however, two passages of OCM 46-3 (4 and 14) and two additional OCMs from 64-3 (separated by EpCam status) were included for RNAseq (36 in total). Of these, 9 were from patients that had not yet received chemotherapy (chemo-naïve), 3 of these patients had an additional OCM generated from a post-treatment sample (biopsy numbers are indicated).

OCMs were established as described in Nelson et al. [27]. Briefly, cells were isolated from ascites by centrifugation, red blood cells removed and remaining cells plated into Primaria flasks containing OCMI [39]. Solid tumour samples were processed using a tumour dissociation kit (Miltenyi Biotec) following the manufacturer’s instructions and cells plated into collagen-coated 12.5-cm^2^ flasks containing OCMI. Cultures were incubated undisturbed for 2–4 days at 37°C in a humidified 5% CO_2_ and 5% O_2_ atmosphere. Media were replaced every 3–4 days. Once attached, stromal cells were separated from the tumour cells using selective trypsinisation. For long-term storage, cells were frozen in Bambanker (Wako pure chemical). OC and stromal cells were subsequently cultured in OCMI media supplemented with 5% FBS (Life Science Group) or 5% Hyclone FBS (GE Healthcare). Cells were passaged at 95% confluence at a ratio of 1:2.

Kuramochi cells for analysis by RNAseq (JCRB Cell Bank) were cultured in RPMI supplemented with 5% FCS, 100 U/ml penicillin, 100 U/ml streptomycin and 2 mM glutamine and were maintained at 37°C in a humidified 5% CO_2_ atmosphere.

### RNASeq of ex vivo ovarian cancer models

RNA was extracted using RNeasy Plus Mini kit (Qiagen), quantified using a Qubit fluorometer (Life Technologies) and quality/integrity assessed using a 2200 TapeStation (Agilent Technologies). Sequencing libraries were then generated using the TruSeq® Stranded mRNA assay (Illumina, Inc.) according to the manufacturer’s protocol. Adapter indices were used to multiplex libraries, which were pooled prior to cluster generation using a cBot instrument (Illumina, Inc.). The loaded flow-cell was then paired-end sequenced (76 + 76 cycles, plus indices) on an Illumina HiSeq4000 instrument. The output data was demultiplexed (allowing one mismatch) and BCL-to-Fastq conversion performed using Illumina’s bcl2fastq software. Note: RNAseq was also performed on a selected number of patient-matched stromal cells, as an additional control, and though this data is not used within these analyses, it is included within the deposited data for completeness.

### RNAseq data processing

The RNAseq data generated by the CCLE [38], and RNAseq of our ex vivo OCMs [27, 40], was processed in the same manner. The paired reads were processed using BBDuk from BBMap v36.32 to trim the adapter sequences and low-quality bases. The filtered reads were mapped to the human reference sequence analysis set (hg38/Dec. 2013/GRCh38) from the UCSC browser, using STAR v2.7.2b [41]. The genome index was created using the comprehensive Gencode v32 gene annotation. The number of reads per gene was counted using ‘--quantMode GeneCounts’ within the STAR command.

### Non-negative matrix factorisation

Data analyses in R were performed using v3.6.2 and Bioconductor v3.10. The DESeq2 (v1.26.0) package was used to apply a variance stabilising transformation to the assembled read count matrix [42]. Transcripts with a median absolute deviation ≥1.5 were selected, and this list of 6796 genes was used as input for clustering analysis using the NMF package [43]. To estimate the factorisation rank (*k*), NMF was performed for *k* of 2 to 10, using 50 random initiation points. Quality measures were computed for each factorisation rank, including the cophenetic coefficients and silhouette width. Inspection of the computed quality metrics revealed two and five clusters fitted the data. Next, 200 iterative runs of NMF were performed from a fixed random initial condition with a *k* value of two and again for a *k* value of five. Using annotations given in the primary literature source for each cell line (Additional file 1: Table S1), we inferred the likely OC histotype of each cluster. Gene scoring schema was applied to extract genes characteristic of the five identified clusters [44]. Metagene lists were combined, and this was used as input for machine learning algorithms.

### Machine learning algorithms for classification

The R package caret (v6.0-86) was used for model training and evaluation. The specific modules used were ‘base::knn’, ‘randomForest’ (v4.6-14) and ‘kernlab’ (v0.9-29), respectively. The subtype assignment gleaned from NMF (*k* = 5; see the ‘Results and discussion’ section) was used to randomly partition cell lines into four groups, such that each subtype was represented in each. Random partitioning was repeated ten times to achieve a reliable estimate of model performance. Each model was trained to each successive set of three groups, and model performance tested on the omitted group. Quality metrics compared between models were the per-subtype sensitivity, specificity and balanced accuracy. Overall model performance was compared using Cohen’s kappa, which compares observed accuracy with the expected accuracy.

### Genetic background of CCLE cell lines

The genetic background of the CCLE cell lines is extensively referred to throughout this manuscript. We direct the reader to the mutation datasets generated by the CCLE. The datasets were originally presented in Ghandi et al. [38] and visualised using the cBioPortal for Cancer Genomics (https://www.cbioportal.org/) that enables interactive exploration of multidimensional cancer genomics datasets [45, 46]. Data is presented as OncoPrint in Fig. 3. For cell line MCAS, a 127-base pair deletion in *TP53* has also been included [28, 47].

### Analysis of primary tumours

Formalin-fixed and paraffin-embedded (FFPE) archival tumour blocks were analysed by immunohistochemistry by collecting 4-μm sections on Superfrost charged slides. After drying overnight at 37°C, samples were processed using a Ventana Benchmark immunohistochemistry platform (Roche) with antibodies against p53 (Dako cat#M700101-2, 1:50), Cytokeratin7 (CK7, Dako cat#M701801-2, 1:250), PAX8 (Roche cat#06523927001, 1:100) and WT-1 (Abcam cat#ab89901, 1:100). Heat-induced epitope retrieval was performed using CC1 (Roche), incubating samples at 95°C for 36, 52, 40 and 64 min for p53, CK7, PAX8 and WT1, respectively. Antibodies were incubated at 37°C for 32, 40, 32 and 40 min for p53, CK7, PAX8, and WT1, respectively. p53 and CK7 were detected using Ultraview universal DAB kit (Roche), while PAX8 and WT1 were detected using Optiview universal DAB kit (Roche), all as per manufacturer’s instructions. Sections were counterstained using Haematoxylin II (Roche) for 12 min and bluing reagent (Roche) for 8 min, and slides imaged using a Leica DM2500 microscope (Leica Microsystems), using a ×20 objective lens under brightfield and processed using Adobe Photoshop. For genotyping, FFPE blocks were assessed for total cellularity and the neoplastic cell content of the sample expressed as a percentage of all nucleated cells on a haematoxylin and eosin (H&E)-stained slide. A neoplastic cell count of ≥10% was required before undertaking DNA extraction. DNA extraction was performed using the cobas® DNA Sample Preparation Kit (Roche). Tumour from 5× 5μM unstained pathology slides was available for DNA extraction. Extracted DNA was quantified using Qubit 2.0 Fluorometer (ThermoScientific). Targeted enrichment was performed using the GeneRead Clinically Relevant Tumour Targeted Panel V2 (Qiagen; *AKT1*, *ALK*, *AR*, *BRAF*, *CTNNB1*, *DDR2*, *EGFR*, *ERBB2*, *FGFR3*, *GNA11*, *GNAQ*, *IDH1*, *IDH2*, *KIT*, *KRAS*, *MAP2K1*, *MET*, *NRAS*, *PDGFRA*, *PIK3CA*, *PTE*N, *RET*, *STK11*, *TP53*). For somatic variants in *TP53*, the target read depth across all coding regions (exon 2 to 9) was a minimum of 350x. Mutations were named according to Human Genome Variation Society guidelines (http://www.hgvs.org/) using reference sequence NM_000546.5. All variant calls were independently reviewed using the BAM files and a genome browser (Integrated Genomic Viewer). At a variant allele frequency ≥ 4%, the call sensitivity was > 90% and specificity > 95% after manual review.

## Results and discussion

### Most frequently utilised CCLE lines are unlikely to be representative of HGSOC

The analyses by Domcke et al. represent an important milestone in the field, utilising in-depth analysis of CNV, mutations and microarray-based mRNA expression profiles to rank 47 OC cell lines according to their resemblance to HGSOC, as defined by comparison with patient samples from TCGA [9, 25]. Therefore, to evaluate cell line usage in recent years, we first performed a literature search including 47 OC cell lines from which the CCLE has recently generated RNAseq data (including 44 of those in Domcke’s analysis) [38]. We counted the number of articles in the literature that refer to each line as an estimate of cell line usage in research, including only the 44 cell lines the search identified as likely epithelial OC (Fig. 1; Additional file 1: Table S1). Seven cell lines collectively constituted almost 90% of OC cell line usage (ranked by most highly used: SK-OV-3, A2780, OVCAR-3, IGROV-1, CAOV-3, 59M and OVCAR-8). Although much of research is focused around HGSOC, only three of these seven lines were scored as highly likely to be HGSOC by Domcke et al. (OVCAR-3, CAOV-3 and 59M). Strikingly, seven cell lines that did score highly as likely to be HGSOC (KURAMOCHI, OVSAHO, SNU-119, COV362, OVCAR-4, COV318 and JHOS-4) only constituted 1% of PubMed citations. Thus, although HGSOC represents the most prevalent subtype, pre-clinical OC research has utilised cell lines that are unlikely to have derived from this subtype. It also remains unclear which histological subtype these frequently used cell lines derive.

### NMF preferentially segregates the OC cancer cell lines into five clusters

**Fig. 2:**
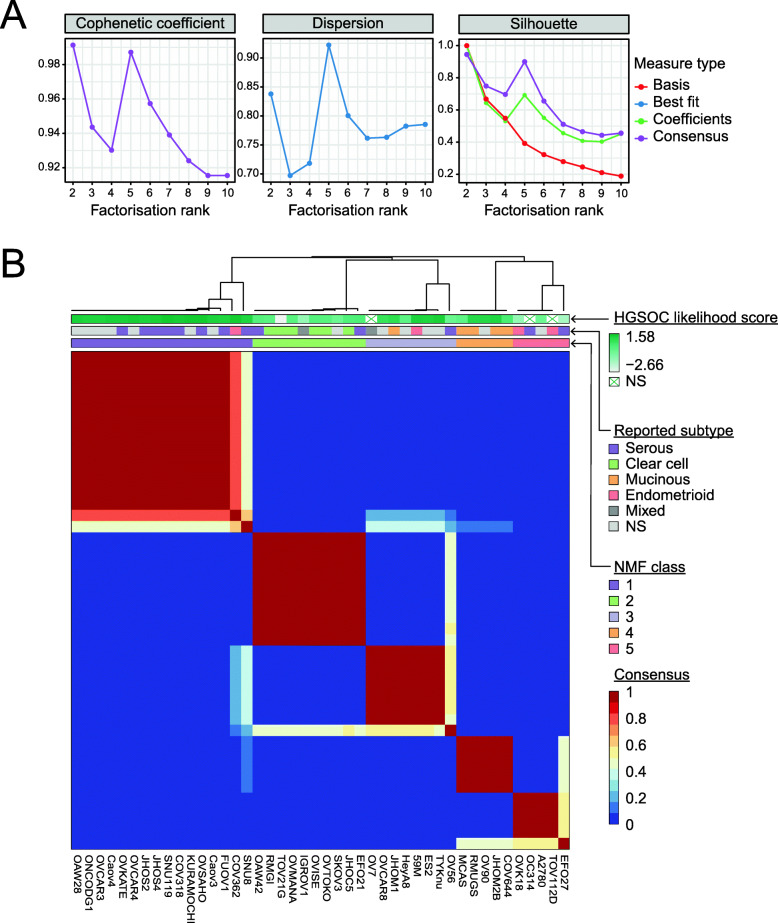
NMF of RNAseq segregates ovarian cancer cell lines into five clusters that recapitulate histological subtypes. **A** Quality metrics describing the performance of NMF for 2 to 10 clusters. From left, the cophenetic correlation coefficients, dispersion and silhouette. Colours indicate the type of measure plotted. **B** Consensus map showing cell line clustering for 200 iterative runs of NMF using 5 clusters. The blocks of the consensus map are coloured by the probability of two samples clustering together. The annotation track atop the heatmap indicates (top) the HGSOC-likelihood score of a cell line determined by Domcke et al. where darker shades represent a higher score; middle, the ovarian cancer subtype provided in the cell line’s original literature source (NS, not specified); bottom, the consensus cluster assignment across 200 NMF runs

We aimed to utilise the RNAseq data from the CCLE [38], in conjunction with NMF, to identify transcriptional signatures specific to each tumour subtype. NMF, as a means of pattern recognition, decomposes overall gene expression into two matrices that approximate it according to the pre-defined number of clusters (*k*). The first matrix defines ‘metagenes’ for each cluster, the small set of genes whose co-expression informs cluster assignment, and the second reflects the co-expression levels of those metagenes in each sample. In order to establish the optimum number of clusters for the cell lines, we first performed NMF at *k* of 2–10 (Additional file 2: Fig. S1). As NMF is generally repeated multiple times using random initiation points to obtain a reliable estimate of classification, we completed 50 NMF runs per *k*. To assess the optimum value of *k*, we considered three quality metrics: the cophenetic correlation coefficient [37], dispersion coefficient [44] and silhouette width [48] (Fig. 2A). Consensus clustering for a five-class split demonstrated high-quality metrics. For *k* = 5, the cophenetic and silhouette width scores were second only to *k* = 2, and the dispersion score was highest for *k* = 5 (Fig. 2A). We subsequently completed 200 NMF runs at *k* = 5 and visualised the data as a consensus matrix, where the entries of the consensus map reflect the probability of two samples clustering together across the multiple NMF runs [49]. Indeed, this generated a consensus map that gave strong evidence for a five-class split, demonstrating a clear block diagonal pattern (Fig. 2B).

To validate optimal NMF clustering at *k* = 5 based on the CCLE dataset, which was generated as part of a high-throughput sequencing project, we repeated the NMF using an independent RNAseq panel from 44 OC cell lines from another pan-cancer study by Klijn et al. [50]. The clustering was markedly similar to that of the CCLE dataset, with clustering at *k* = 5 demonstrating good quality metrics (Additional file 2: Fig. S2). In addition, for 29 cell lines common between these datasets, clustering was mirrored in both consensus maps (Fig. 2B and Additional file 2: Fig. S2). Interestingly, this confirmed the clustering of OV56 with other known CCOC cell lines, as it had a low silhouette score in our original NMF, but a high silhouette score using the alternative dataset. Indeed, our group has noted differences between CCLE profiling and gene expression levels measured by Nanostring for this cell line [51]. In general, samples clustered with lower silhouette scores into *k* = 5 with the alternative versus the CCLE dataset, potentially due to the absence of non-coding transcript levels in the Ensembl-annotated dataset from Klijn et al. Hence, it could be inferred that expression of long non-coding RNAs play a role in fine-tuning of the distinct clusters. Nevertheless, this independent validation confirms the fidelity of the transcriptional profiles generated by the CCLE and concurs that NMF optimally segregates frequently used OC cell lines into five clusters based upon transcriptional profiling.

### The five NMF clusters represent the five main subtypes of epithelial OC

**Fig. 3:**
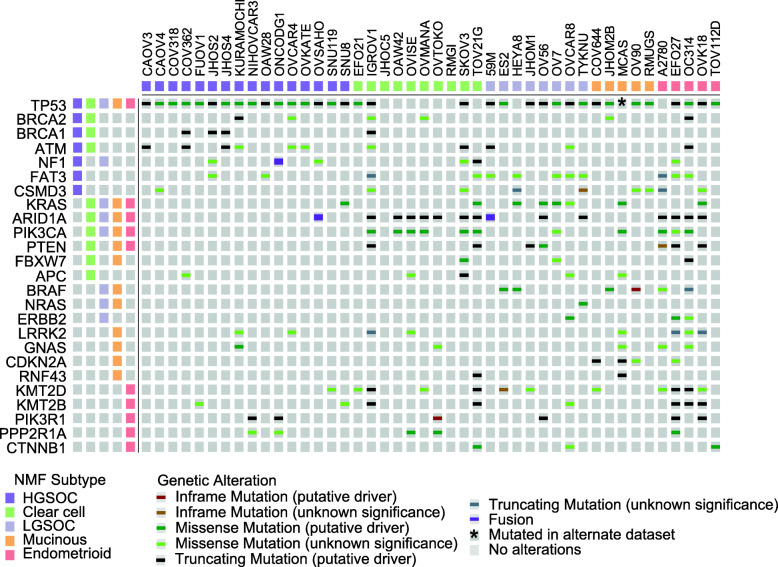
Mutational landscape of identified clusters is concordant with that of clinical cohorts. Gene mutations in the five different subtypes of epithelial OC were identified from the literature (Additional file 1: Table S3 and references therein). Mutations in these genes were determined using the Cbioportal and visualised as an oncoprint diagram. Note: MCAS was shown separately to harbour a 127 base pair deletion in *TP53* [28]. The track along the top indicates the subtype as identified by NMF of transcriptional profiling (Fig. 2). To the left, the tracks indicate whether a mutation has been identified in a cohort of patient samples of that subtype (see Additional file 1 for references)

As NMF preferentially segregated the OC cell lines into five clusters, we considered whether these clusters represented the five main subtypes of epithelial OC. We first examined the subtype assigned by the primary literature source for each cell line, where this was available (Additional file 1: Table S1 and references therein). Indeed, this showed a clear overrepresentation of cell lines annotated with a given subtype within each cluster at *k* = 5, suggesting that the clusters from left to right in Fig. 2B represent HGSOC, CCOC, LGSOC, MOC and ENOC. We subsequently confirmed these putative assignments by comparing the mutational profiles of the cell lines with those from corresponding clinical cohorts (Fig. 3 and below).

#### High-grade serous ovarian cancer models

HGSOC is the most common histological subtype, characterised by aggressive dissemination. Although most patients respond to primary treatment, nearly all patients with advanced stage disease will relapse, at which point cure is highly unlikely [52]. The precursor of a substantial proportion of HGSOC is likely to be serous tubal intraepithelial carcinoma (STIC) in the fimbriae of the fallopian tubes [53–55]. Indeed, STIC harbour the same *TP53* mutations as the surrounding invasive carcinoma, suggesting a clonal relationship [56–58]. *TP53* is mutated in >96% of HGSOC cases, and histopathological review of wild-type tumours suggests *TP53* alterations are likely ubiquitous [9, 59–61]. Half of the cases also display HR deficiency, most frequently due to mutations in *BRCA1/2* (~20%) or *BRCA1* promoter methylation (~11%) [8–10, 60, 62]. CNV is extensively observed, with amplifications frequently involving oncogenes, such as *MYC* (31.5%), *CCNE1* (21.7%) and *PIK3CA* (18%), and deletions involving tumour suppressor genes, such as *PTEN* (6.1%) [9, 45, 46].

Of the 16 OC cell lines in the first cluster of the NMF consensus map, eight were assigned as ‘serous’ in their primary literature annotation, and seven were not specified (Fig. 2B; dark purple track). To confirm putative identification of this cluster as HGSOC-derived cell lines, we aligned the cell lines that cluster here with those identified as HGSOC by Domcke et al. (Fig. 2B; blue/green graduated track). All 16 cell lines are among the top 20 scoring cell lines in Domcke’s analysis, strongly supporting the transcriptional classification by NMF. Furthermore, mutational profiling of the cell lines in this cluster revealed mutations characteristic of HGSOC, including *TP53* (100% of cell lines) and *BRCA1/2* (31.25% of cell lines) (Fig. 3). Thus, this provides assurance that eight cell lines (OVSAHO, SNU-119, COV318, JHOS-4, JHOS-2, OVKATE, FU-OV-1 and SNU8) transcriptionally and genetically resemble the tumour subtype they were reported to derive from. However, SNU8, which was ranked 20th by Domcke et al., falls into cluster 3 (LGSOC) in 40% of NMF initialisations. Also, while it does harbour a *TP53* mutation, *KRAS* is also mutated, an event that is rare in HGSOC [9]. Thus, SNU8 is an unusual cell line with features of both HGSOC and LGSOC that cannot be resolved by genetic or transcriptional profiling.

One additional cell line that both NMF and Domcke et al. support to be HGSOC is COV362, which was originally designated as ENOC in its primary literature source [63]. Indeed, the WHO note the difficulty in distinguishing high-grade ENOC from HGSOC, in line with the possibility that the original tumour may have been misclassified [3]. However, like SNU8, COV362 also shows a low silhouette score across 200 runs of NMF, also clustering 25% of the time into cluster 3 (LGSOC), suggesting that it may share some characteristics with these cell lines. Importantly, COV362 does not cluster in any NMF run with other cell lines reported as ENOC. Furthermore, it has *TP53* and *BRCA2* mutations that are characteristic of HGSOC (Fig. 3). Finally, seven cell lines without specified subtype in their primary literature source were confirmed to represent models of HGSOC based on transcriptional clustering (KURAMOCHI, OVCAR-4, Caov-4, OAW28, Caov-3, ONCO-DG-1 and OVCAR-3), supporting previous analysis by Domcke et al.

#### Clear cell ovarian cancer models

CCOC is the second most prevalent subtype of epithelial OC, with the highest frequency reported in Asian countries, whereby it accounts for up to 30% of cases in Japan but only 10% in Europe and North America [64, 65]. Although CCOC more frequently presents at an early stage and in younger women [21, 64, 66], when adjusted for stage survival, rates are worse for CCOC than HGSOC [21, 66]. The most common mutations in CCOC include *PIK3CA* (~50.0%), *ARID1A* (~45%), *TP53* (~20%) and *KRAS* (~10%) [13, 67]. Unlike the origin-specific transcriptional profiles of serous or endometrioid tumours, clear cell tumours have a particularly distinct transcriptional profile that is maintained across clear cell carcinomas of the ovary, endometrium and kidney [68].

The second cluster of our NMF consensus map is enriched for known CCOC cell lines (Fig. 2B; green track). Of the cell lines in this cluster, six were originally annotated as CCOC, two as serous, one as mixed adenocarcinoma and one was not specified; no cell line annotated as CCOC clustered separately. These cell lines have a high frequency of mutations that have been identified in patients with CCOC, including in *ARID1A* (7 of 10), *PIK3CA* (6 of 10), *TP53* (3 of 10) and *KRAS* (1 of 10; Fig. 3). Four cell lines also had mutations in *KMT2D*, which has been reported in a CCOC case [69] and more recently found to be mutated in a significant number of ENOC cases (8 of 26; 31%) [14]. The two cell lines in this cluster originally annotated as serous, EFO21 and OAW42, received relatively low HGSOC-likelihood scores in the analysis by Domcke et al. In addition, unlike almost all HGSOC [9, 59], OAW42 is *TP53* wild-type; however, it does harbour two frameshift mutations within *ARID1A* (Fig. 3), supporting its designation as CCOC [67]. Also, EFO21 has a lower fraction of the genome altered than cell lines that cluster with our purported HGSOC lines.

The remaining two cell lines that fall into the CCOC cluster are SK-OV-3 and IGROV1. Although both are frequently assumed to be serous in origin, SK-OV-3 was originally described as simply ‘adenocarcinoma of the ovary’, and IGROV1 as mixed adenocarcinoma containing endometrioid, serous, clear cell and undifferentiated components. Indeed, both had a low HGSOC-likelihood score from Domcke et al. Rather, the mutational landscape of SK-OV-3 coincides with three of the most commonly mutated genes in CCOC: *PIK3CA*, *ARID1A* and *TP53* (Fig. 3) [13, 67]. Furthermore, SK-OV-3-injected mice formed clear cell adenocarcinomas [70]. Interestingly, Domcke et al. reported IGROV1 as unlikely to be HGSOC as it is hypermutated, and instead suggested it to be of ENOC or CCOC origin due to its clustering with endometrium-derived cancer cell lines by microarray-derived transcriptional profiling [9, 25]. The assumption that IGROV1 is of serous origin possibly stems from the presence of mutations within *TP53* and *BRCA1/2* (Fig. 3); however, IGROV1 has mutations in *ARID1A*, *PIK3CA* and *PTEN*, which appear to be exclusive to non-HGSOC subtypes (Fig. 3). This highlights the unique benefit of using transcriptional profiling to aid in the diagnosis of epithelial OC subtypes, as genetic mutations can occur within multiple different subtypes [71].

Both our NMF and the immunohistochemistry panel by Anglesio et al. [28] placed JHOC5, TOV21G, OVTOKO and OVMANA as CCOC lines. Other lines we identify as CCOC were classified as atypical non-serous or were not classified by Anglesio et al. [28]. Interestingly, all of the HNF1B-positive lines in the study by Anglesio et al. are designated CCOC by NMF, with the exception of OV90, which also stains positive. Positive HNF1B staining has been shown to be almost ubiquitous in CCOC, with significantly higher frequency than in HGSOC and ENOC [72–74].

#### A potential low-grade serous ovarian cancer cluster

LGSOC accounts for only ~3% of epithelial OC and was until recently described as grade 1 serous or well-differentiated serous adenocarcinoma [7]. LGSOC is distinct from HGSOC, with younger age at presentation, differing pathological and molecular characteristics, less aggressive behaviour and longer overall survival [3, 75, 76]. However, LGSOC are typically chemotherapy-resistant and suboptimal surgical debulking leads to similar outcomes to HGSOC [16, 77, 78]. LGSOC arise from serous cystadenoma or adenofibroma, which progresses through serous borderline tumour to invasive carcinoma in a slow stepwise manner [3]. LGSOC often harbour activating mutations of genes involved in the MAPK signalling pathway, including *KRAS* (~20–35%), *BRAF* (~10–40%), *ERBB2* (~5%) and *NRAS* (~10%) [16, 17, 79–82]. Mutations in key MAPK pathway genes are mutually exclusive, meaning one of these genes is mutated in around half to two-thirds of LGSOC [17, 79, 80]. *TP53* mutations are rare in LGSOC, ranging from 0–8% prevalence across studies, though some series use the absence of *TP53* mutations as an inclusion criterion [16–18].

As LGSOC represents a fairly recent descriptor, it is more difficult to infer this classification from literature annotations. Furthermore, 4 of the 8 cell lines in the third cluster were not designated a subtype in their primary literature source. However, we propose that the third cluster of the NMF consensus map may represent LGSOC (Fig. 2B; light purple track). In keeping with the frequency of MAPK pathway mutations in LGSOC, cell lines in this cluster harbour the highest frequency of *KRAS* mutations (4 of 8) and additionally show *BRAF* (2 of 8), *NRAS* (1 of 8) and *ERBB2* (1 of 8) mutations; in fact, 7 of 8 cell lines have a mutation in at least one of these genes. However, none of these cell lines harboured mutations in *USP9X*, which has recently been found at a high frequency in LGSOC cases [17]. Also, OV-56 more likely represents CCOC, as described above, based on alternative datasets [50, 51]. Nevertheless, cluster three closely mimics the genetic landscape of LGSOC and this designation potentially identifies 4 cell lines previously unspecified in the literature as LGSOC (TYK-nu, HeyA8, ES2 and OVCAR8). In addition, OV7, which was previously described as mixed adenocarcinoma, is also identified to be representative of LGSOC.

This putative LGSOC cluster contains three cell lines with a top 20 HGSOC-likely score and one ranked ‘possibly-HGSOC’ by Domcke et al.: TYK-nu, 59M and ES2, and JHOM-1, respectively. In agreement with LGSOC designation, TYK-nu has two mutations in *NRAS*. Additionally, 59M (previously annotated as ENOC) has three mutations in MAPK pathway proteins, and ES2 (previously subtype unspecified) has a *BRAF* mutation, and they are both therefore characteristic of LGSOC [16, 79]. While JHOM-1 does not harbour a MAPK pathway mutation, it does have fewer CNV and point mutations than the cell lines designated HGSOC [38]. Although these four cell lines also harbour *TP53* mutations, an overrepresentation of *TP53*-mutated cell lines relative to the proportion in respective tumour type has been reported previously [83], which may be due to selective pressure for a *TP53* mutant clone during ex vivo expansion. Indeed, it is difficult to establish cell lines from low-grade, slow-growing indolent tumours [84].

Interestingly, proteomic profiling by Coscia et al. also found 59M and TYK-nu to be distinct from other HGSOC cell lines [25, 30]. While the proteomic signature of one group of cell lines closely resembled both HGSOC and cultured fallopian tube epithelial cells, the group containing 59M and TYK-nu resembled that of immortalised ovarian surface epithelial cells. The authors therefore suggest that heterogeneity exists in the proteome of HGSOC based on disparate sites of origin [30], and indeed, there are recent reports that a subset of HGSOC are of ovarian surface epithelium origin [85–87]. However, segregation may reflect the differences between HGSOC- and LGSOC-derived cell lines. Indeed, based on the frequency of MAPK pathway mutations in these cell lines, which are extremely rare in HGSOC, we assign this NMF cluster as likely LGSOC in origin in our analysis.

A cell culture and morphology-based study by Beaufort et al. identified three different morphologies of epithelial OC lines: epithelial, round and spindle, which showed distinct biological and molecular characteristics [29]. Interestingly, the three cell lines we purport to be LGSOC, which are in common between our two studies (OV7, 59M and ES2), all demonstrated spindle-like morphology. They all also demonstrated low or absent EpCAM staining and tended to have the lowest doubling times of the cell line panel, suggesting that these features may be characteristic of LGSOC [29].

#### Mucinous ovarian cancer models

MOC are morphologically characterised by epithelium with intestinal differentiation, and thus, it can be challenging to determine whether a disease is primary ovarian or a secondary mucinous adenocarcinoma that originated elsewhere. Historically, many mucinous tumours involving the ovary were in fact metastases from extra-ovarian sites and, after revisions to the diagnostic criteria, the rate of MOC fell from ~10% to only 3% of epithelial OC [88, 89]. Note therefore that this study is not designed to determine a non-ovarian origin of purported MOC cell lines. MOC is diagnosed at stage 1 in 80% of cases, when the prognosis following surgery is good. However, advanced stage disease has a poor prognosis, due to low response rates to platinum-based therapies [90]. Genetic analyses of primary MOC support a progressive model of carcinogenesis, whereby benign cystadenoma develops a *KRAS* or *CDKN2A* mutation, progressing to borderline tumours likely to have both events and additional CNV, to overt carcinoma, which display a higher frequency of *KRAS* and *TP53* mutations (both ~60% in MOC), and greater CNV [11, 91, 92]. CNVs are key cancer drivers associated with increasing grade and metastatic progression [11]. Other mutations identified in MOC include *RNF43*, *BRAF*, *PIK3CA* and *ARID1A* (8–12%), as well as amplification of *ERBB2* (26%) [11].

Of five OC cell lines annotated in their primary reference as MOC, four cluster together (Fig. 2B; orange track). These are MCAS, RMUG-S, COV644 and JHOM-2B. OV-90 also clusters with the MOC cell lines, which originally was not designated a subtype. In support of designation as MOC, OV-90 harbour *ERBB2* amplification, and *BRAF* and *TP53* mutations (Fig. 3) [11, 93]. JHOM-2B was in the top 20 HGSOC-likely cell lines defined by Domcke et al.; however, it is reported in the literature as MOC, and our NMF also clusters it with other MOC cell lines. In fact, Domcke et al. ranked JHOM-2B as 19th, close to the threshold for designation as only ‘possibly HGSOC’. Indeed, this cell line does harbour a *TP53* mutation (Fig. 3); however, *TP53* mutations are also present in around 60% of MOC [11, 12]. The fifth cell line reported as MOC in its original publication, but excluded from this cluster, is JHOM-1, which falls into the cluster we tentatively class as LGSOC and has been discussed previously.

Anglesio et al. found TFF3 mRNA, a marker that is significantly more highly expressed in mucinous carcinoma, was detectable in two cell lines within their panel, MCAS and OV-90 [28], consistent with our placement of these two cell lines as MOC. However, in the Anglesio study, OV-90 was not classified by their algorithm due to an almost equal call of endometrioid and HGSOC. Targeted sequencing by Anglesio et al. did not assess *BRAF* or *CDK2NA*, mutations characteristic of MOC, that were identified by the CCLE supporting placement of MCAS and OV-90 by NMF here as MOC.

#### Endometrioid ovarian cancer models

ENOC can have a more favourable prognosis than HGSOC, as it tends to present at an earlier stage and at a younger age [94, 95]. The most common gene mutations associated with ENOC include *CTNNB1* (25–53%), *PTEN* (17–46%), *KRAS* (33–42%), *PIK3CA* (27–40%), *ARID1A* (19–30%), *KMT2D* (31%), *KMT2B* (19%) and *TP53* (7–19%) [14, 15, 96]. A subset of ENOC closely resembling HGSOC, with *TP53* mutations, HR deficiency and widespread CNV, was also recently identified [14, 97]. Indeed, high-grade or extensive mucinous differentiation in ENOC may be difficult to differentiate from HGSOC and MOC, respectively, based upon morphological features alone [3, 98]. Indeed, ENOC was the most frequently reclassified histologic type in biomarker-assisted reviews of OC series [99, 100].

Accordingly, cell lines purported to represent ENOC fall into multiple clusters but are concentrated within the final NMF cluster (TOV112D and OVK18; Fig. 2B; red track). Two other cell lines with a primary annotation of ENOC, 59M and COV362, segregate into the clusters designated LGSOC and HGSOC. All five cell lines within the ENOC cluster collectively display a mutational profile in line with ENOC tumours: specifically, mutations in *TP53* (4 of 5), *ARID1A* (4 of 5), *KMT2D* (4 of 5), *PIK3CA* (3 of 5), *PTEN* (3 of 5), *KMT2B* (3 of 5) and *KRAS* (1 of 5; Fig. 3) [14, 15, 96]. A2780, which is newly annotated as a model of ENOC, displays mutations in *ARID1A*, *PIK3CA*, *PTEN* and *KMT2D* (Fig. 3).

EFO27 and OC314, which are assigned to the ENOC cluster, were originally classified as serous. However, both harbour *ARID1A* and *PIK3CA* mutations, among other mutations common with ENOC (Fig. 3). While OC314 had not been molecularly characterised at the time, EFO27 also received a low HGSOC-likelihood score from Domcke et al. [9, 25]. Therefore, the genetic similarities between these cell lines, and the reported lack of HGSOC features, suggest they are more accurate models of ENOC. However, it should be noted that EFO27 has a poor silhouette score in our consensus map (Fig. 2B), clustering with other ENOC cell lines in 58% of NMF runs, and with MOC cell lines in the other NMF runs, suggesting it shares transcriptional features with both subtypes.

Our NMF clustering suggests that a hypermutated genotype is common among ENOC and CCOC. Three of five hypermutated cell lines (high mutation frequency with few CNVs) fall into the ENOC cluster (EFO27, OVK18 and OC314) and the remaining two (TOV21G and IGROV1) fall into the CCOC cluster. Indeed, mismatch repair deficiency, which leads to a hypermutated genotype, has been exclusively identified in low-grade, low-stage ENOC and CCOC (18% and 2%, respectively), in keeping with Lynch syndrome-associated ovarian cancer [101, 102]. Therefore, this further supports designation of these five hypermutated OC lines as of ENOC or CCOC origin.

### Dualistic model of ovarian carcinogenesis may be oversimplistic

An alternate, dualistic model of ovarian carcinogenesis (types I and II) has been proposed to consolidate the clinical presentation of OC subtypes with their molecular characteristics [5, 6]. The type I class, which includes LGSOC, ENOC, CCOC and MOC, is described as including characteristically low-grade, indolent tumours, with frequent alterations in cell signalling pathways [5, 6]. Type II tumours include mostly HGSOC and are described as aggressively growing tumours, with near-ubiquitous *TP53* mutation and chromosome instability. This model was acknowledged by the WHO classification in 2014, with LGSOC and HGSOC described as the prototypical type I and II tumours, respectively [3]. As NMF also supported a case for a two-cluster fit (Fig. 2A and Additional file 2: Fig. S1A), this poses the question as to whether these two clusters are representative of the dualistic classification.

To ascertain whether the NMF clustering into two groups reflects the dualistic model, we annotated the cell lines within the two clusters with the subtypes defined by NMF at *k* = 5. Indeed, all LGSOC-labelled cell lines fell into cluster 1, and all HGSOC-labelled cell lines fell into cluster 2, with the exception of SNU8, which had a poor silhouette score at *k* = 5; (Additional file 2: Fig. S1A; Fisher’s exact test, *p* ≤ 0.001). Given that LGSOC and HGSOC are the prototypic type I and II tumours, the molecular and clinical features that gave rise to the dualistic classification are mirrored within the transcriptional profiles of the corresponding cell lines. However, in addition to LGSOC—ENOC, CCOC and MOC are generally considered type I tumours [5, 6]. Interestingly, we found that non-serous-labelled cell lines were split between the two clusters. Therefore, we conclude that the two clusters identified by NMF here do not represent type I and II tumours as described by the dualistic model.

As type II tumours have been described as predominantly *TP53*-mutated, we assessed whether *TP53* status could explain the differences between the two clusters. There was a trend towards a higher proportion of *TP53*-mutated cell lines present in cluster 2 (Fisher’s exact test, *p* = 0.15), suggesting that *TP53* status may be influencing clustering. Furthermore, data suggests no enrichment of chemo-naive or chemotherapy-treated lines in either cluster (Fisher’s exact test; *p* = 0.4018). However, as treatment history is infrequently given in the original publication that established these cell lines, we cannot rule out a relationship based on available data. Likewise, based on the cell lines annotated with the site of biopsy, those sampled from the ovary or ascites were not enriched in either cluster (Fisher’s exact test; *p* = 0.934).

In line with our results, it has been questioned whether a dualistic model of OC is reflective of the heterogeneity of so-called type I tumours [103]. Even within histological subtypes, this group is not homogenous. Indeed, CCOC has been suggested to belong to an intermediate, rather than a type I, category [104]. Furthermore, as mentioned above, a subset of aggressive ENOC cases closely resembling HGSOC has been identified, with *TP53* mutations, HR deficiency and widespread CNVs [14, 97]. Although these may represent HGSOC cases with a ‘pseudoendometrioid pattern’ [5, 105], our clustering using *k* = 5 suggests they are a distinct and ENOC-derived subset, while demonstrating similarity to HGSOC.

There are limitations to drawing conclusions on the classification of OC from clustering performed on OC cell lines. Namely, ‘type II-like’ type I cancers may possess an inherent growth advantage and greater ability to adapt to culture conditions than their more indolent counterparts. Meaning, aggressive tumours could constitute a minority of clinical cases but are highly represented among cell lines. It is also possible that the two clusters reflect tissue of origin; however, as the cell-of-origin for some subtypes remains unclear, RNAseq data from tissue from these potential sites would be required to test this. For example, while both CCOC and ENOC are well-known to be endometriosis-associated, cell-of-origin is controversial with proposed sources including endometrium, endometrial cysts, ovarian surface epithelia and fallopian tube-derived cells [106]. Despite these possibilities, our finding that non-serous cell lines fall into both NMF clusters at *k* = 2 mirrors concerns that the spectrum of these histotypes is oversimplified by a dualistic model [103]. However, our analysis does highlight some merits of the dualistic model, confirming the stratification of serous tumours into exclusive high-grade and low-grade-containing groups.

### Training of a machine learning classifier to predict ovarian cancer subtype

Mutation profiling of the CCLE cell lines within the five NMF clusters supports our histotype designation of each cluster and informs the selection of the most appropriate CCLE cell lines representing all five main subtypes of epithelial OC. However, clonal selection in long-term culture means that cell lines are unlikely to display the true heterogeneity of primary tumours. For example, although single-cell-derived colorectal cancer cell lines display ongoing random instability, a specific karyotype is maintained over time [32]. Consequently, OC researchers are developing living biobanks of patient-derived samples, which have the potential to more accurately predict patient response to therapeutics [27, 33]. Hence, we considered whether transcriptional profiling could be used to support histological subtype assignment of patient-derived ex vivo ovarian cancer models (OCMs). We aimed to develop such a ‘transcriptional classifier’ by trialling machine learning algorithms on the RNAseq profiles of the newly annotated CCLE cell lines.

**Fig. 4:**
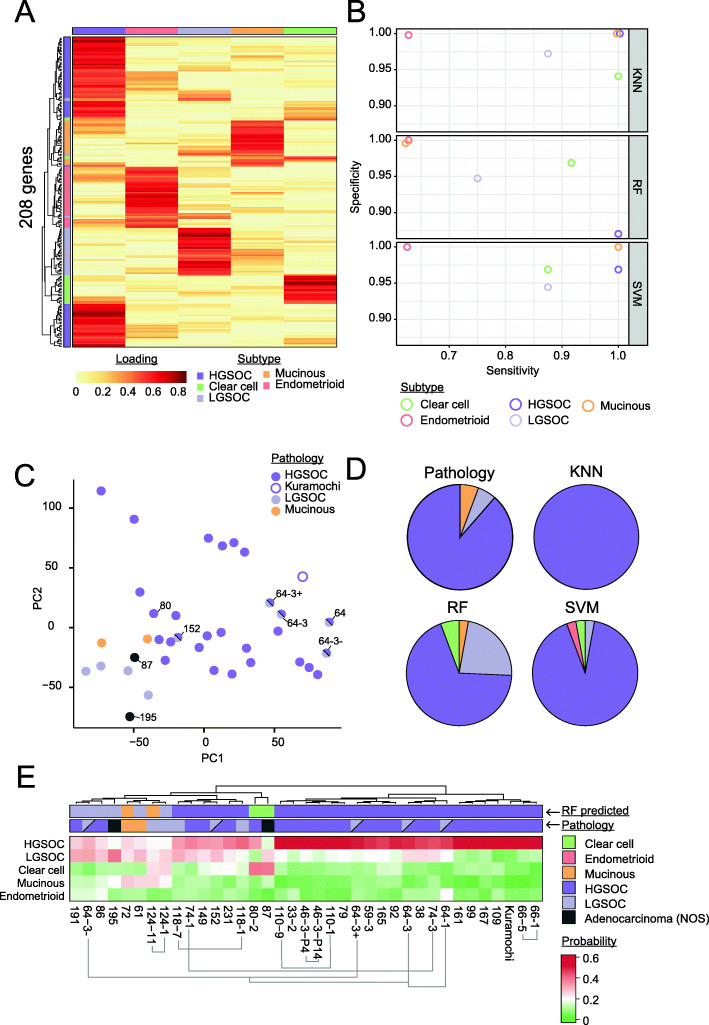
Ability of a k-nearest neighbour classifier to predict subtype of ovarian cancer cell lines. **A** Metagene signatures for which high expression is informative of each cluster were extracted using gene scoring scheme as per Kim and Park [44]. Colours represent the strength of the association between that gene and the cluster, where red indicates the strongest association. The top track indicates cluster number, as per Fig. 2. **B** Evaluation of three machine learning algorithms for OC cell line subtype classification: k-nearest neighbour (KNN), random forest (RF) and support vector machine (SVM). Cell lines were designated the subtype indicated by NMF clustering and partitioned into 4 subsets. Three subsets were used to train each of the machine learning algorithms, with the fourth set held out as a test set. The four subsets were rotated such that each sample had the opportunity to be trained and tested upon. The average per-class sensitivity and specificity score across the four tested sets are shown. Balanced accuracy scores for HGSOC were 1 (KNN), 0.935275 (RF) and 0.984375 (SVM), and the overall kappa values for each model are 0.918 (KNN), 0.78905 (RF) and 0.878 (SVM). **C** Principal component analysis of patient-derived OCMs. Colours indicate the subtype determined by a pathologist. **D** Comparison of the identified subtype based upon pathology, and the k-nearest neighbour (KNN), random forest (RF) and support vector machine models trained in B deployed on the OCMs. **E** Closer inspection of the performance of the RF model. Pathology and RF-predicted subtype are indicated above the heatmap. HGSOC cell line Kuramochi is included in parts **C**–**D** as a positive control. The models are referred to using the OCM prefix followed by the patient number and, if one of a series, the biopsy number. + EpCAM positive; − EpCAM negative; P4 and P14 indicate passage number of this OCM; NOS, not otherwise specified

To initially test the potential of a transcriptional classifier, we determined whether the NMF classification of the CCLE cell lines could be used to train a machine learning model to predict the subtype of a ‘hold-out’ set of the lines. Genes with expression levels characteristic for each cluster were first extracted, and the list combined and used to train the models. The largest number of genes was associated with the HGSOC cluster (82 genes), followed by ENOC (40 genes), LGSOC (35 genes), MOC (28 genes) and CCOC (23 genes; Fig. 4A). The classification potential of three trained models (k-nearest neighbour [KNN], random forest [RF] and support vector machine [SVM]) was next evaluated by comparison of per-subtype specificity and sensitivity metrics (Fig. 4B). All models strongly predicted HGSOC, achieving balanced accuracy scores of 1 (KNN), 0.94 (RF) and 0.98 (SVM), presumably reflecting the large number of HGSOC cell lines and associated genes. Therefore, inclusion of additional non-HGSOC cell lines would greatly aid the training of a classifier; for example, ENOC is only represented by 5 of the 44 cell lines included in this study. Nevertheless, the overall kappa values achieved for each model were 0.92 (KNN), 0.79 (RF) and 0.88 (SVM).

### Deployment of classifiers to predict subtype of ovarian cancer models

Having established that the trained classifiers can accurately predict the subtype of held-out CCLE cell lines, we next deployed them on patient-derived OCMs from our living biobank [27]. The OCMs are clinically annotated with histotype, allowing comparison of classifier prediction with the histological diagnosis (Additional file 1: Table S2). The OCMs demonstrate karyotypic heterogeneity characteristic of OC, while being unfettered by contaminating, wild-type stromal cells and the tumour microenvironment, and therefore represent an important step in the evaluation of a transcriptional classifier [27]. The OCMs also provide an indication of classifier utility as they underwent RNAseq independently of the CCLE cell lines on which the classifiers were trained. At the time of analysis, 36 OCMs, from 27 patients, had RNAseq data available [27, 40]. Based on histology assessment, this cohort was predominantly from HGSOC, with four OCMs derived from LGSOC (OCM.118-1, and 118-7, OCM.124-1 and 124-11), two MOC (OCM.72 and OCM.61), two from patients with a cytological diagnosis of ‘suspicion of adenocarcinoma arising from the gynaecological tract’ (OCM.87 and OCM.195), and one from a moderately differentiated (intermediate grade; grade 2) serous adenocarcinoma (OCM.152) (Additional file 2: Fig. S3). These ‘non-HGSOC’ OCMs cluster closely by principal component analysis (PCA), supporting the potential of transcriptional profiling in differentiating subtypes (Fig. 4C). Finally, although OCMs 64-1 and 64-3 were diagnosed as LGSOC, there is evidence to suggest mixed histology associated with these OCMs and they cluster with HGSOC by PCA (see below; Fig. 4C) [27].

For deployment of machine learning models on the OCMs, the KNN, RF and SVM classifiers were trained using the complete set of CCLE cell lines. Despite performing best in terms of overall kappa in predicting cell line subtype, the KNN model predicted all of the OCMs to be HGSOC (Fig. 4D). This may indicate overtraining due to the high number of genes relative to number of samples trained on (208 metagenes versus 44 cell lines). Likewise, the SVM model predicted the majority of OCMs to be HGSOC. However, RF classifier prediction more closely aligned with histology (Fig. 4D, E). This classifier correctly assigned 76% of the 29 OCMs with unambiguous histology (Fig. 4E). Of the non-HGSOC OCMs, 72 and 124-1 were correctly designated MOC and LGSOC, respectively. However, for non-HGSOC subtypes, there was generally a more even-split of trees voting for each subtype. This lower performance in predicting specific non-HGSOC subtype is in agreement with the lower per-class sensitivity and specificity observed for these subtypes during training (Fig. 4B).

### Integration of case histories with molecular features

Local (historical) and central (re-analysed in this study) histology assessment disagreed for patients 118 and 124, with the final diagnosis confirmed as LGSOC in both. The RF classifier prediction assigns both OCM.124-1 and OCM.124-11 as non-HGSOC therefore supports the revised pathology. While the classifier predicts OCM.124-1 to be LGSOC, the prediction of OCM.124-11 as MOC may reflect lower performance in predicting non-HGSOC subtypes, or possibly molecular alterations that have occurred following treatment (Fig. 4E). However, in the case of OCM.118-1 and 118-7, both were predicted as HGSOC by the RF classifier. Case note review of patient 118 did not reveal any details suggestive of an alternate diagnosis, as the patient exhibited clinical hallmarks of LGSOC, including minimal response to primary platinum-based chemotherapy (Additional file 2: Fig. S3A), wild-type p53 staining and strongly diffuse PR staining (data not shown). Wild-type *TP53* status of both OCMs 118-1 and 118-7 was also confirmed by immunofluorescence on response to nutlin-3 treatment (data not shown). However, although the classifier also predicts OCM.118-7 to be HGSOC, a larger proportion of decision trees predicted LGSOC for the later sample than the earlier sample (Fig. 4E; columns of the heatmap), suggesting there may be a mixed population of tumour cells, or a response to treatment.

As previously stated, the OCMs derived from patient 64 may also have originated from a mixed population of cells, since we previously showed that OCM.64-3 can be divided into two main populations based upon EpCAM status [27]. These two populations, OCM.64-3^Ep−^ and OCM.64-3^Ep+^, are designated LGSOC and HGSOC by the RF classifier, respectively, though they were expanded from the same patient sample. The classifier also predicted OCM.64-1, derived from an earlier sample, to be HGSOC. In line with a HGSOC, OCMs 64-1, OCM.64-3^Ep−^ and OCM.64-3^Ep+^ all have an identical *TP53* mutation [27]. However, panel-based next-generation sequencing (NGS) on the primary tumour block and exome sequencing of the OCM [27] demonstrated a mutation in *KRAS*, consistent with the high frequency of this mutation in LGSOC [16, 17, 79–82]. Indeed, while the local (primary) pathology diagnosis reported HGSOC for this tumour [27], a review by an expert gynaecological pathologist (S.D.) suggested that the tumour more closely resembles LGSOC overall, with low-grade cytological atypia and low mitotic activity. Moreover, although predominantly heterogenous (wild-type) p53 immunohistochemistry staining was present, focal areas of strong staining were also evident, suggestive of two potential populations of cells (Additional file 2: Fig. S3B). Thus, transcriptional classification may be helpful in assisting pathological assessment in equivocal cases.

For the two OCMs with pathology given only as ‘suspicious of adenocarcinoma arising from the gynaecological tract’, the RF classifier predicted OCM.87 to be CCOC and OCM.195 to be LGSOC, both with high probability, highlighting the utility of such a classifier when only ascites is available for a cytological diagnosis. Note that we previously concluded from the patient’s case notes that OCM.87 was HGSOC [27]; however, our up-to-date review suggests a clinical diagnosis of ‘suspicious of adenocarcinoma arising from the gynaecological tract’ is more appropriate. By PCA, OCM.87 clustered closely with other ‘non-HGSOC’ OCMs (Fig. 4C). In addition, exome sequencing of this OCM suggests it resembles CCOC, rather than HGSOC, as it is *TP53* wild-type, but does display a highly elevated mutational load, possibly indicating a tumour driven by a mismatch repair defect as it harbours an *MLH1* mutation [27]. This is in line with previous reports finding microsatellite instability in CCOC and ENOC [101, 102]. Furthermore, clinical review finds that this patient presented with relatively low CA-125 (77 IU/ml) and a paraneoplastic syndrome; both more indicative of CCOC than HGSOC [3]. Finally, the RF classifier strongly predicts OCM.195 to be LGSOC-derived. Pathology review identified clinical features consistent with a diagnosis of LGSOC, including minimal response to primary platinum-based chemotherapy (Additional file 2: Fig. S3A). Our sequencing panel also demonstrated a mutation in *KRAS*, consistent with the high frequency of mutation in this gene in LGSOC [16, 17, 79–82]. No *TP53* mutation was detected and the OCM demonstrated a functional p53 response to nutlin-3 treatment by immunofluorescence (data not shown).

Overall, our data demonstrate the potential of a transcriptional classifier as a tool for subtype validation of novel epithelial OC models, identification of atypical clinical presentations, and for classification of new models when clinical annotation is unavailable or if a definitive pathology-based diagnosis is not possible. Inclusion of additional cell lines would improve predictive performance, especially of subtypes that are underrepresented in the CCLE dataset. Alternatively, the classifier may be improved by repeating the NMF clustering on a larger biobank of ex vivo cultures, to optimise the classifier gene sets for these cultures and culture conditions. Additionally, datasets containing patient-derived cell lines could be utilised to further evaluate performance, including expansion of our living biobank and others [27, 39, 107].

## Conclusions

Classification of disease subtype is important both for clinical decision-making and for selection of appropriate model systems for pre-clinical research into different disease entities. Although it is widely accepted that epithelial OC is a heterogenous disease with five main subtypes, selection of appropriate models representative of each of these subtypes remains a significant challenge for research [25, 28–30]. Previous studies aimed to address this challenge by defining an immunohistochemical, genetic or combinatorial panel and determining the suitability of cell lines to fit this mould. Conversely, we did not impose any prior knowledge or structure onto RNAseq data, instead opting to use NMF, a clustering algorithm that has also been used for other pattern-recognition problems such as facial recognition [37, 108]. Transcriptional profiling using NMF classified the OC cell lines into five clusters, and the mutational landscape of the cell lines provides strong evidence that these clusters represent the five main histological subtypes. Our analysis therefore now informs selection of CCLE cell lines as models for research on all five main subtypes of epithelial OC.

Attempts to refine OC subtype disease classification include the dualistic model of type I and II tumours [5, 6]. Our analysis supports previous concerns that CCOC, ENOC and MOC are distinct and do not conform to a simple dualistic type I classification [103]. Our results do, however, confirm the stratification of serous tumours into exclusive HGSOC and LGSOC groups. We have also demonstrated the promise of a ‘transcriptional classifier’ developed by using machine learning approaches that, with optimisation, could be utilised both for subtype validation of novel models and for supporting classification of new models when clinical annotation is unavailable. Our results support the potential value of such a classifier in providing confidence that appropriate subtype models are being utilised in research; however, wider use for disease classification could also be envisioned following further research, for example, where diagnosis is uncertain, to aid stratification of patients into clinical trials for targeted therapy and to ensure accurate histopathological diagnosis. Translation of this classifier into a diagnostic biomarker will require testing both on a larger biobank, with adequate representation of all subtypes, as well as on RNAseq from complex patient samples with varying tumour heterogeneity.

## Supplementary Information

**Additional file 1.** Table S1 (Cell line annotations); Table S2 (Patient demographics); Table S3 (References for subtype mutations).

**Additional file 2 **Fig. S1 (Consensus cluster maps for NMF at different values of k), Fig. S2 (NMF at k=5 using RNAseq from OC cell lines from study by Klijn *et al*.); Fig. S3 (Clinical review of selected OCMs).

## Data Availability

The RNAseq dataset from 19 novel OCMs is available from EBML-EBI using accession number E-MTAB-10801 [40] and FASTQ files are available from European Nucleotide Archive (https://www.ebi.ac.uk/ena/browser/view/PRJEB46736). An R script to perform RF classification of new ovarian cancer models is available at https://github.com/bethmbarnes/RF-prediction-of-ovarian-cancer-subtype [109]. Additional RNAseq datasets used in this study have been published previously: Raw RNAseq sequence reads from the CCLE are available from European Nucleotide Archive: PRJNA523380 [38]. RNAseq from Klijn et al. (E-MTAB-2607) [50] and from 17 additional OCMs (E-MTAB-7223) [27] are also available from EBML-EBI.

## Abbreviations

CCLE: Cancer Cell Line Encyclopedia
CCOC: Clear cell ovarian cancer
ENOC: Endometroid ovarian cancer
HGSOC: High-grade serous ovarian cancer
HR: Homologous recombination
KNN: k-nearest neighbour
LGSOC: Low-grade serous ovarian cancer
MOC: Mucinous ovarian cancer
NGS: Next-generation sequencing
OC: Ovarian cancer
OCM: Ovarian cancer model
PCA: Principal component analysis
RF: Random forest
SVM: Support vector machine
TCGA: The Cancer Genome Atlas
