# Supplementary material for: Distinct transcriptional programs stratify ovarian cancer cell lines into the five major histological subtypes
Source: Genome Med. 2021 Sep 1;13:140. doi: 10.1186/s13073-021-00952-5 (PMC8408985; doi:10.1186/s13073-021-00952-5)
Supplement: Supplementary file 2 — Additional file 2 Fig. S1 (Consensus cluster maps for NMF at different values of k), Fig. S2 (NMF at k=5 using RNAseq from OC cell lines from study by Klijn et al.); Fig. S3 (Clinical review of selected OCMs). [file 13073_2021_952_MOESM2_ESM.pdf]

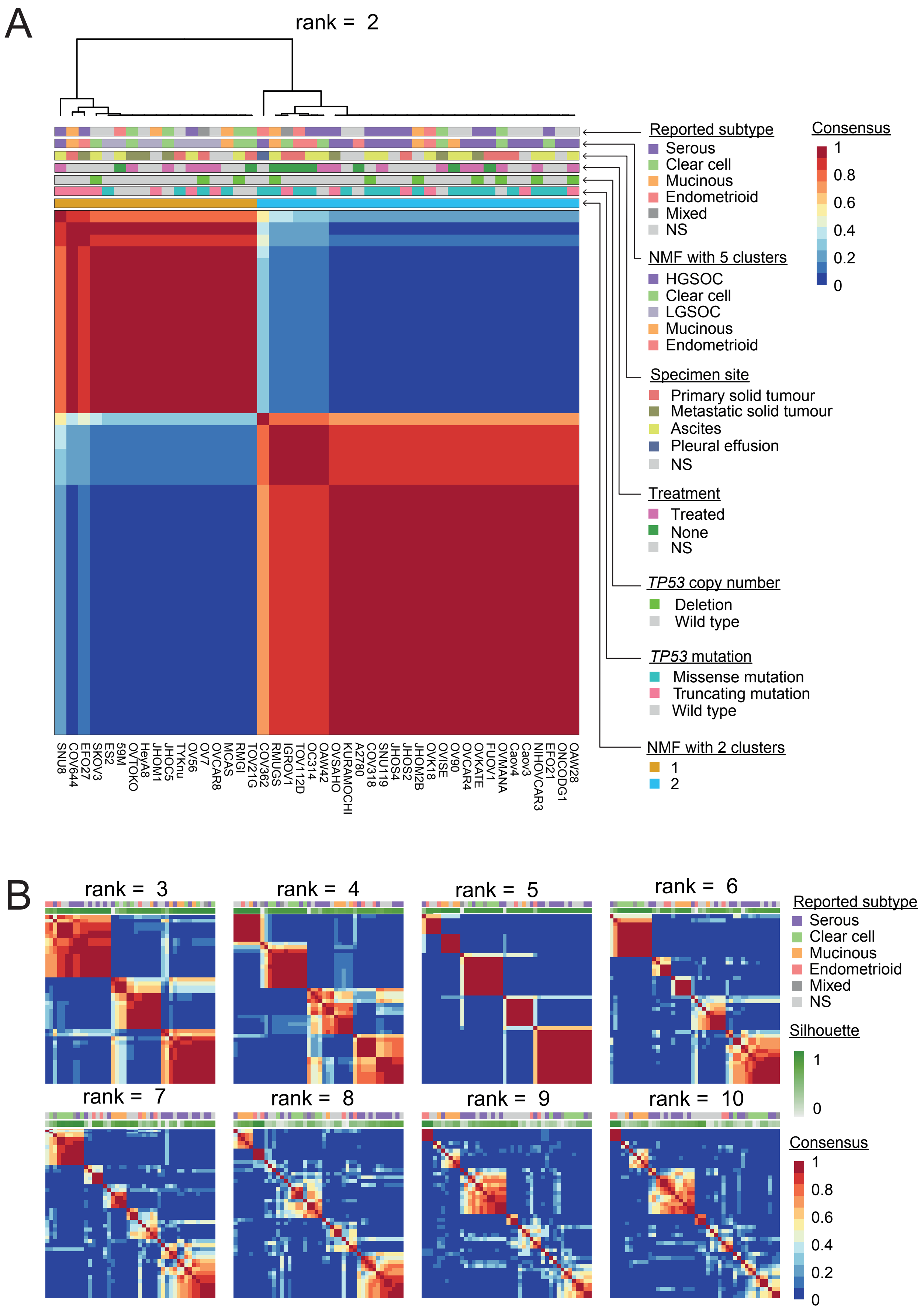
A
rank = 2
Reported subtype
Serous
Clear cell
Mucinous
Endometrioid
Mixed
NS
NMF with 5 clusters
HGSOC
Clear cell
LGSOC
Mucinous
Endometrioid
Specimen site
Primary solid tumour
Metastatic solid tumour
Ascites
Pleural effusion
NS
Treatment
Treated
None
NS
TP53 copy number
Deletion
Wild type
TP53 mutation
Missense mutation
Truncating mutation
Wild type
NMF with 2 clusters
1
2
Consensus
1
0.8
0.6
0.4
0.2
0
SNU8
COV644
EFO27
SKOV3
ES2
59M
OVTOKO
HeyA8
JHOM1
JHOC5
TYKnu
OV56
OV7
OVCAR8
MCAS
RMGI
TOV21G
COV362
RMUGS
IGROV1
TOV112D
OC314
OAW42
OVSAHO
KURAMOCHI
A2780
COV318
SNU119
JHOS4
JHOS2
JHOM2B
OVK18
OVISE
OV90
OVCAR4
OVKATE
FUOV1
OVMANA
Caov4
Caov3
NIHOVCAR3
EFO21
ONCODG1
OAW28
B
rank = 3
rank = 4
rank = 5
rank = 6
rank = 7
rank = 8
rank = 9
rank = 10
Reported subtype
Serous
Clear cell
Mucinous
Endometrioid
Mixed
NS
Silhouette
1
0
Consensus
1
0.8
0.6
0.4
0.2
0

Figure S1

Fig S1. Consensus cluster maps for NMF at different values of k. (**A**) Consensus cluster map for k=2 clusters. The tracks above the map, from top to bottom, indicate the reported subtype of each cell line in its primary literature source, the designation based on NMF for k=5 clusters (see text), specimen site, treatment status, the *TP53* status based upon copy number analysis, the *TP53* status based upon mutational profiling, and the stratification of cell line based on NMF using k=2 clusters. (**B**) Consensus cluster maps from 3 to 10 clusters (for k=2, see A). The blocks of the consensus map are coloured by the probability of two samples clustering together. The annotation tracks atop the heatmap indicate the OC subtype provided in the cell line's original literature source where green=CCOC; red=ENOC; orange=MOC; purple=serous; dark grey=mixed; light grey=not specified (NS). Bottom track, silhouette width for each sample pair where dark green indicates a silhouette width of 1 (perfect clustering).

A

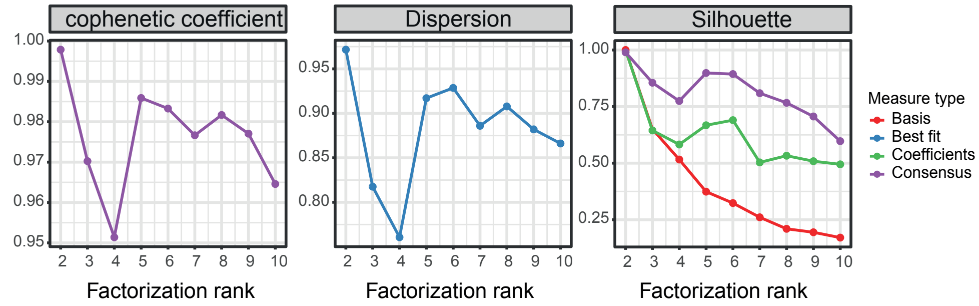
cophenetic coefficient
Dispersion
Silhouette
Factorization rank
Factorization rank
Factorization rank
Measure type
Basis
Best fit
Coefficients
Consensus

B

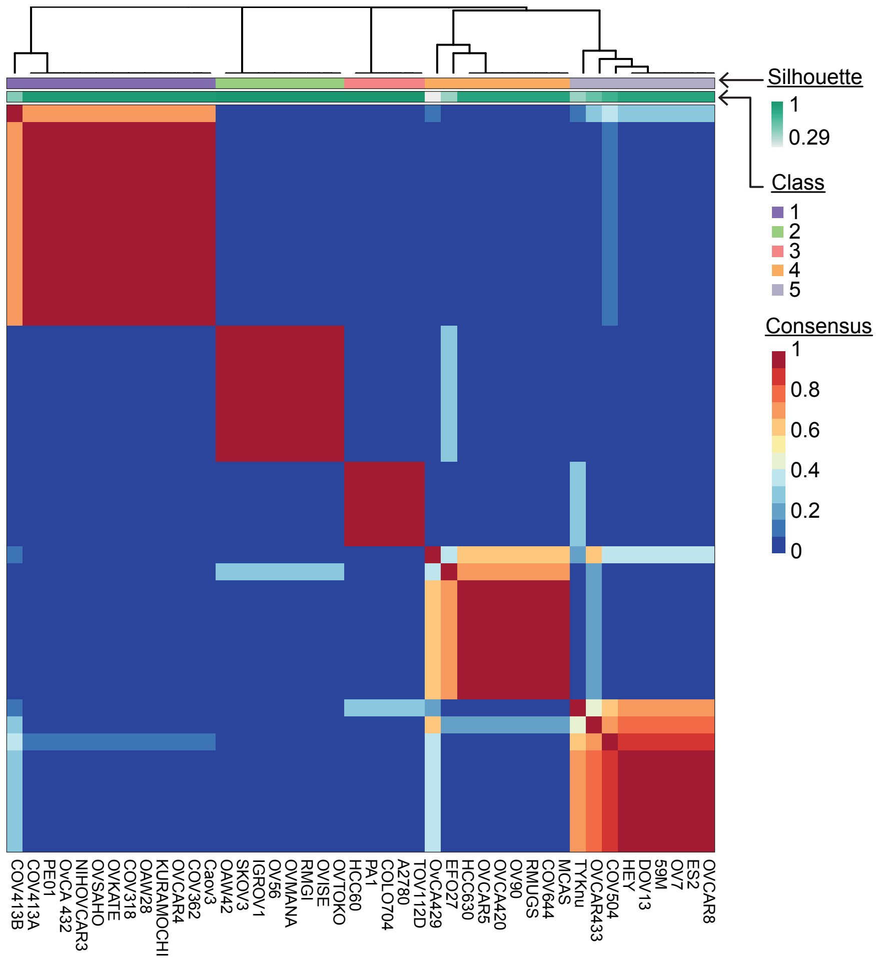
Silhouette
1
0.29
Class
1
2
3
4
5
Consensus
1
0.8
0.6
0.4
0.2
0
COV413B
COV413A
PE01
OvCA 432
NIHOVCAR3
OVSAHO
OVKATE
COV318
OAW28
KURAMOCHI
OVCAR4
COV362
Caov3
OAW42
SKOV3
IGROV1
OV56
OVMANA
RMGI
OVISE
OVTOKO
HCC60
PA1
COLO704
A2780
TOV112D
OvCA429
EFO27
HCC630
OVCAR5
OvCA420
OV90
RMUGS
COV644
MCAS
TYKnu
OVCAR433
COV504
HEY
DOV13
59M
OV7
ES2
OVCAR8

Figure S2

Fig S2. NMF at k=5 using RNAseq from OC cell lines from study by Klijn *et al.* (**A**) Quality metrics describing the performance of NMF for 2 to 10 clusters. From left, the cophenetic correlation coefficients, dispersion and silhouette. Colours indicate the type of measure plotted. (**B**) Consensus map showing cell line clustering for 200 iterative runs of NMF using 5 clusters. The blocks of the consensus map are coloured by the probability of two samples clustering together, where red=1; white=0.5 and blue=0. The annotation tracks atop the heatmap indicate the consensus cluster assignment across the 200 NMF runs where dark purple=cluster 1; green=cluster 2; light purple=cluster 3; orange=cluster 4 and red=cluster 5. Bottom track, the silhouette score of each cell line where darker shades represent a higher score.

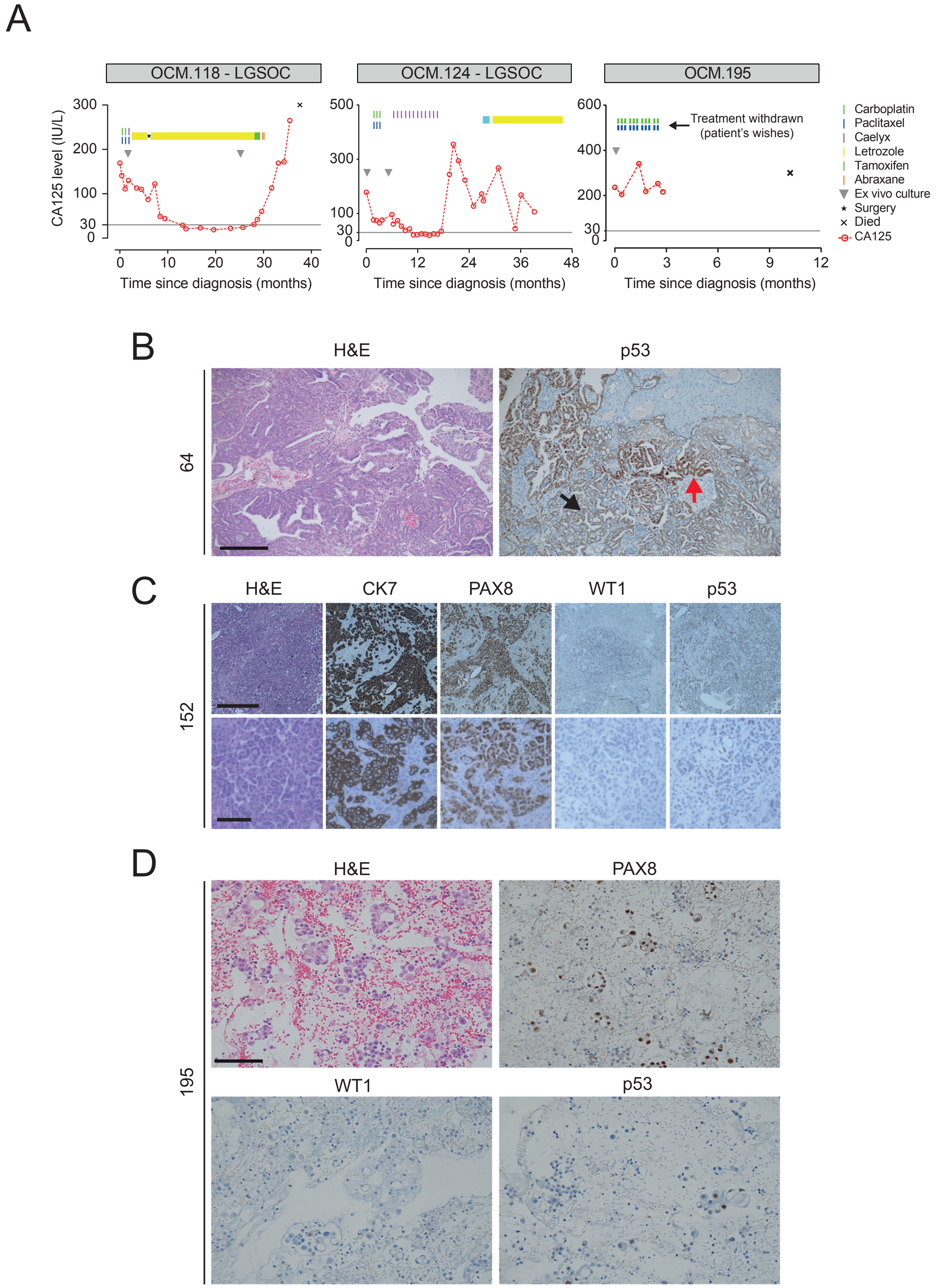
A
OCM.118 - LGSOC
OCM.124 - LGSOC
OCM.195
CA125 level (IU/L)
Time since diagnosis (months)
Time since diagnosis (months)
Time since diagnosis (months)
Treatment withdrawn (patient's wishes)
Carboplatin
Paclitaxel
Caelyx
Letrozole
Tamoxifen
Abraxane
Ex vivo culture
Surgery
Died
CA125
B
H&E
p53
64
C
H&E
CK7
PAX8
WT1
p53
152
D
H&E
PAX8
WT1
p53
195

Figure S3

Fig S3. Clinical review of selected OCMs. (**A**) Graphical representation of changes in serum CA-125 level throughout the course of illness for the patients from whom OCMs 118, 124 and 195 were derived. Graphs show lines of chemotherapy prior to ascites sample (*ex vivo* culture). The upper limit of normal of CA-125 was 30 IU/mL (grey straight line). (**B–D**) Representative images of H&E, CK7, PAX8, WT1 and p53 immunohistochemistry staining from primary tumour block (**B**) Black arrow indicates p53 wildtype IHC staining, while Red arrow indicates p53 focal strong (mutant-type) staining. Scale bar: 100 μm (x20 magnification). (**C**) Upper panel (x10 magnification) scale bar: 500 μM, low panel (x40 magnification) scale bar: 100 μM. (**D**) This cytology block contained a mixture of tumour and mesothelial cells, which makes interpretation of immunostaining difficult as these two cell types cannot separately be identified. Scale bar: 100 μm (x20 magnification).
